# Turning over new ideas in human skeletal muscle proteostasis: What do we know and where to from here?

**DOI:** 10.1113/EP092353

**Published:** 2025-02-05

**Authors:** Changhyun Lim, James McKendry, Matthew Lees, Philip J. Atherton, Nicholas A. Burd, Andrew M. Holwerda, Luc J. C. van Loon, Chris McGlory, Cameron J. Mitchell, Kenneth Smith, Daniel J. Wilkinson, Tanner Stokes, Stuart M. Phillips

**Affiliations:** ^1^ Exercise Metabolism Research Group, Department of Kinesiology McMaster University Hamilton Ontario Canada; ^2^ Population Health Sciences Institute, Faculty of Medical Sciences Newcastle University Newcastle upon Tyne UK; ^3^ Food, Nutrition and Health, Faculty of Land and Food Systems The University of British Columbia Vancouver BC Canada; ^4^ MRC/ARUK Centre for Musculoskeletal Ageing Research and National Institute of Health Research, Biomedical Research Centre, School of Medicine University of Nottingham Derby UK; ^5^ Ritsumeikan Advanced Research Academy (RARA) Fellow and Visiting Professor, Faculty of Sport and Health Science Ritsumeikan University Kyoto Japan; ^6^ Department of Health and Kinesiology and Division of Nutritional Sciences University of Illinois Urbana‐Champaign Urbana Illinois USA; ^7^ Department of Human Biology, NUTRIM Institute of Nutrition and Translational Research in Metabolism Maastricht University Medical Center+ Maastricht the Netherlands; ^8^ School of Kinesiology and Health Studies Queen's University Kingston Ontario Canada; ^9^ Department of Medicine Queen's University Kingston Ontario Canada; ^10^ Faculty of Education, School of Kinesiology The University of British Columbia Vancouver BC Canada; ^11^ Department of Sport and Exercise Sciences Manchester Metropolitan University Institute of Sport Manchester UK

**Keywords:** deuterium oxide, human, phenotype, skeletal muscle, stable isotope tracer

## Abstract

Understanding the turnover of proteins in tissues gives information as to how external stimuli result in phenotypic change. Nowhere is such phenotypic change more conspicuous than skeletal muscle, which can be effectively remodelled by increased loading, ageing and unloading (disuse), all of which are subject to modification by nutrition and other environmental stimuli. The understanding of muscle proteome remodelling has undergone a renaissance recently with the reintroduction of deuterated water (D_2_O) and its ingestion to label amino acids and measure their incorporation into proteins. However, there is confusion around the use of the deuterated water methodology and the interpretation of the data it provides. Here, we provide a short review of some of the more salient features of the method and clarify some of the confusion around the method of deuterated water methods and its use in humans and how the interpretation of the data is in contrast to that of rodents.

## Abstract (highlights)

- **What is the topic of this review?**
  The deuterated water methodology and its application in measuring human tissue, with a focus on skeletal muscle protein turnover.
- **What advances does it highlight?**
  Ingestion of deuterated water is being increasingly used to examine how external stimuli – feeding, loading, exercise and ageing – affect skeletal muscle protein turnover. We update the reader on important advances in this methodology and its limitations and dispel misconceptions about its use.

## 1 INTRODUCTION

Advances in analytical mass spectrometry have opened up new avenues for utilising stable isotope tracers to enhance our comprehension of human physiology (Wilkinson, 2018). Effectively deploying stable isotope tracers requires an understanding of the physiological processes being studied, as well as the principles and assumptions underlying the use of these tracers. In human metabolic research, methods such as stable isotope infusions (e.g., flooding dose and primed‐constant substrate‐specific tracer infusions) and the resurgence of deuterated water (D_2_O) have enhanced our understanding of human skeletal muscle protein turnover (proteostasis) and the various factors influencing it, including exercise, ageing, nutrition and disease. The D_2_O approach permits measurements of muscle protein synthesis (MPS) over days, weeks or even months, delivering a marked change in our understanding of the acute to chronic regulation of MPS. For the interested reader, further methodological details of the D_2_O approach in humans are discussed in our recent review (Holwerda et al., 2024). While substrate‐specific tracer infusions have provided invaluable information as to the acute responses of MPS (and muscle protein breakdown, MPB) to nutrition, exercise and hormones, these responses do not incorporate anabolic and catabolic stimuli that may fluctuate over days to weeks and may not always relate to longer‐term outcomes. That said, both acute and chronic tracer approaches can be used within a single study to provide a greater mechanistic (acute infusion) and closer‐to‐phenotypic (D_2_O ingestion) understanding of the regulation of proteostasis.

It has always been imperative to critically appraise the frameworks through which researchers investigate questions related to muscle proteostasis using both substrate‐specific and ‘substrate agnostic’ (D_2_O) tracing. Here, our group of experts, who have made extensive use of these methodologies, lay out a short review of the methodologies and their application in humans, as well as future avenues, for tissue protein turnover. We hope that this short review will aid researchers who are planning on applying some of the methodologies we outline, particularly the use of a deuterated water tracer in humans for measuring tissue, particularly muscle, protein synthesis.

## 2 PROTEOSTASIS

Proteostasis is crucial for maintaining an efficiently functioning proteome that supports cellular metabolism and overall organismal health. It involves the coordinated action of protein synthesis and degradation in a steady state and during acute and chronic metabolic perturbation (e.g., fasting, feeding, exercise, ageing and disease) that, over time, leads to changes in the proteome and presumably phenotype. While we posit that fluctuations in MPS are largely responsible for changes in myofibrillar protein mass, MPB is also important. For instance, protein degradation avoids the accumulation of damaged, dysfunctional and misfolded proteins. This intricacy is highlighted by the relationship between the immune system and proteostatic mechanisms (i.e., immunoproteasome) and subsequent cellular function (Ruano, 2021). As such, interventions seeking to inhibit or mitigate protein breakdown are likely to be indiscriminate in the proteins they target, and we do not know what the longer‐term consequences of suppressing protein breakdown would be. Therefore, it is unlikely that the suppression of protein breakdown can yield favourable outcomes (Stokes et al., 2018). The notion of MPS–good and MPB–bad is not consummate with new understandings in skeletal muscle remodelling. However, if proteins targeted for degradation are damaged – misfolded, oxidised, nitrosylated – then prevention of their clearance is analogous to not taking out the rubbish from a system, and so would seem a poor strategy to a greater net positive protein balance to promote retention of skeletal muscle protein mass. Indeed, there are numerous pathologies in which muscle proteolysis is impaired, leading to defective muscle phenotypes.

## 3 MUSCLE PROTEIN SYNTHESIS: BULK AND INDIVIDUAL PROTEIN TURNOVER APPROACHES

**FIGURE 1:**
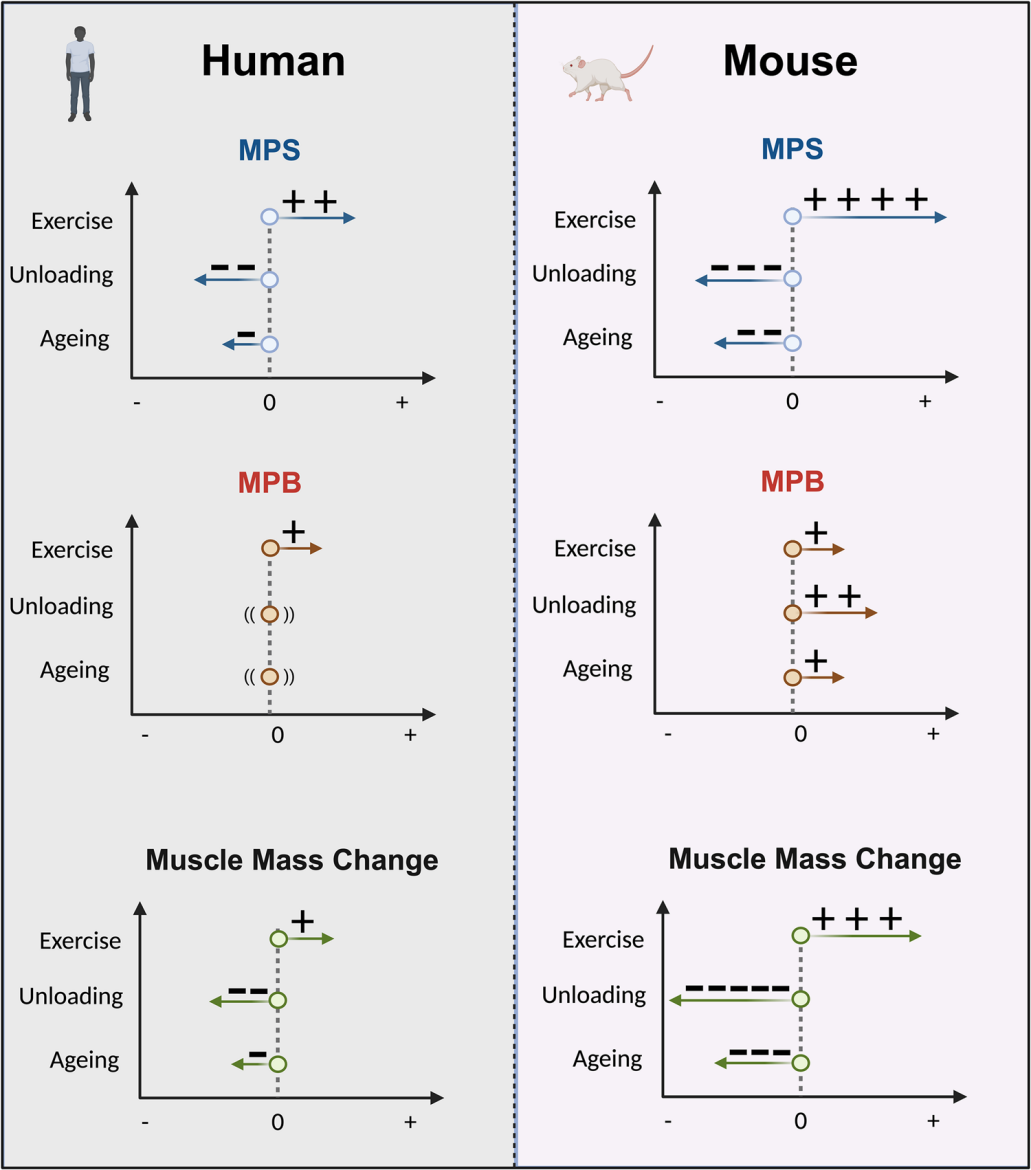
Changes in protein synthesis, breakdown and muscle mass in response to exercise (loading), unloading and ageing in humans and mice. Schematic representations of the relative change in muscle mass comparing humans to mice. In human studies, MPB is minimally or not altered in response to exercise, unloading and ageing, with changes in MPS primarily responsible for alterations in muscle mass under these conditions. In contrast, mouse studies show increased MPB or the expression of genes related to protein degradation following unloading models, such as hindlimb suspension and denervation. Thus, although measuring MPB is also crucial, focusing on alterations in MPS could be a reasonable approach to determining the proteostasis response to these conditions in humans. Created with BioRender.com.

From a methodological perspective, far more published human studies have investigated the change in muscle mass in response to exercise, nutrition, disuse and ageing and assessed MPS. The assessment of MPS is relatively straightforward compared to measuring MPB (Stokes et al., 2018). Infusing or ingesting a tracer over hours or days/weeks, respectively, and assessing the change in the incorporation (i.e., baseline to the time point of interest) of the tracer label into bound muscle protein and particular subfractions (e.g., myofibrillar, sarcoplasmic or mitochondrial) is relatively simple. The complexity of assessing MPB, including arteriovenous catheters, prior labelling of protein pools (which is often costly and can obscure temporal resolution), and concerns about tracer recycling have meant far fewer investigations of the MPB ‘side’ of the proteostasis equation. These limitations have also led to the use of static biomarkers as proxies for bulk (i.e., relating to explaining physiological change) proteolysis. Crucially, while MPB should not be ignored, ample evidence shows that changes in protein synthesis (mixed and myofibrillar) are primarily responsible for changes in muscle mass in response to resistance exercise (McKendry et al., 2021), ageing (Shad et al., 2016) and disuse (Phillips & McGlory, 2014) in humans (Figure 1).

Nevertheless, examining proteostasis of the human extracellular matrix (ECM) provides an example of the potential for altered breakdown being responsible for protein accumulation. Indeed, given the accumulation of collagenous protein depositions in the ECM of older individuals (Nederveen et al., 2020), it is plausible that degradation‐resistant proteins/aggregates may accumulate within the muscle ECM. In contrast, there are no data in humans to suggest that specific proteins in the myofibrillar or sarcoplasmic fractions are ‘resistant’ to turnover. If part of the myofibrillar fraction were resistant to degradation, this would not impact the synthesis rates of other proteins to maintain bulk fractional synthesis. Indeed, from an energetics perspective, while protein synthesis is among the most energetically expensive processes in the cell and energy resources are limited, an increase in the synthesis of one protein does not necessarily lead to a decrease in the synthesis of another. Proteostasis does not imply that the sum of all proteins in bulk protein synthesis remains constant. Thus, it is incorrect to view bulk protein turnover as a zero‐sum game: the term ‘steady state’ refers to how a single variable (e.g., MPS and MPB herein) stays constant while consuming energy. It does *not* relate to the net balance between protein synthesis and degradation. To exemplify, if both MPS and MPB are equal and opposite (which must only be transient), there can exist multiple *distinct* steady states of lower or higher turnover, which may have gross effects on physiology in the absence of a theoretical alteration in net balance or an incorrectly assumed steady state.

Given the relative ease with which MPS can be determined, there is a tendency to conflate the simplicity of assessment with the value of information yielded. Advanced techniques like D_2_O‐based dynamic proteomics may offer deeper insights into individual protein turnover, including the ability to discriminate between fast and slow turnover of proteins (Camera et al., 2017). However, traditional methods provide valuable temporal information in controlled settings, particularly when combined with measures of pool size. Indeed, we have shown in a longitudinal study that MPS measured using D_2_O over the first 3 weeks of a study is equivalent to MPS over the second 3 weeks in an untrained limb (Brook et al., 2015). Any such influence of slower versus faster turning over proteins on bulk protein turnover must, therefore, be negligible. There is a greater likelihood that faster turning over proteins are more represented in measures over more acute periods (i.e., <24 h) as they may initially dominate labelling in mixed muscle protein and thus ‘skew’ the data. Nonetheless, the pool size of such proteins versus the slower turnover pools (much akin to the contribution of non‐muscle proteins to bulk measures) may also be important. Yet, assuming both non‐muscle cell fractions and the most quickly turning over pools (within a biopsy extraction) represent a small proportion of total protein, the overall impact on interpretation from bulk measures must be questioned.

In subsequent sections, we address how loading, ageing and unloading affect skeletal muscle proteostasis in humans and finally provide a perspective on tracer considerations in crucial comparison to animal models.

## 4 WHAT DO WE KNOW ABOUT AGEING AND HOW IT AFFECTS PROTEOSTASIS?

The ageing process has a substantial impact on the skeletal muscle proteome (McKendry et al., 2021). In contrast to disease and disuse, the progressive loss of muscle mass with age occurs at a much slower rate (Nunes et al., 2022). As a consequence of this more prolonged time course of muscle loss, it is far more difficult to determine which process, either MPS or MPB (or both), is driving the loss. Nevertheless, an intrinsic aspect of ageing skeletal muscle that sets it apart from disuse and disease is a sustained loss of proteostasis (Nunes et al., 2022; Paez et al., 2023). In the postabsorptive state, the rates of MPS and MPB do not appear to differ with advancing age, and the findings on age‐related postprandial differences in MPB are inconsistent (Shad et al., 2016). The progression of sarcopenia is proposed to largely be a product of a desensitised MPS response to anabolic stimuli, or anabolic resistance, such as loading and/or protein ingestion, in ageing (Shad et al., 2016).

The loss of proteostasis with age could be fibre‐type specific, as revealed by advanced single‐fibre proteomics (Murgia, Toniolo et al., 2017). Type I myofibres of older adults show an increased expression of proteins that are involved in protein synthesis (i.e., ribosomal and elongation factor proteins) and protein degradation (i.e., proteasomal subunits and autophagic regulatory proteins) alongside chaperone proteins that serve to maintain protein structure (McKendry et al., 2021; Murgia, Toniolo et al., 2017; Nunes et al., 2022). In contrast, type II myofibres demonstrate the decreased expression of these proteins, indicating impaired maintenance of sarcomeric integrity and stress‐buffering capacity in type II fibres. These fibre‐specific characteristics of the ageing proteome might explain, at least in part, the failed reinnervation of aged skeletal muscle that appears to be a feature of primarily type II fibres (McKendry et al., 2021). These molecular differences between fibre types might also partially explain why type I fibres are seemingly well‐preserved with age, whereas type II fibres show significant atrophy.

The expression of mitochondrial genes (Phillips et al., 2013) and proteins (Murgia, Toniolo et al., 2017) is significantly decreased in older skeletal muscle. A recent investigation showed that around 25% of mitochondrial proteins were shown to have altered abundance in ageing, with the majority (∼70%; 173 proteins) being downregulated (Ubaida‐Mohien et al., 2019). Of these downregulated proteins, 16 were components of respiratory chain complexes (complex I–V), which aligns with the impaired mitochondrial respiratory function observed in muscle fibres from older adults (Murgia, Toniolo et al., 2017). Concomitant with the loss in mitochondrial protein content, type I fibres seem to increase components of the glycolytic machinery; however, this is not the case for type II fibres (McKendry et al., 2021; Murgia, Toniolo et al., 2017; Nunes et al., 2022). Impairments in mitochondrial function are also accompanied by an increase in proteins that promote fat storage, for example, perilipin 1 (PLIN1) and tissue inhibitor of metalloproteinase 3 (TIMP3), which might mechanistically underpin the increased intramuscular fat reported in older adults (McKendry et al., 2021). The greater abundance of TIMP3 might also contribute to the degradation of the ECM as well as collagen deposition in aged muscle (Alameddine & Morgan, 2016; McKendry et al., 2021). It is noteworthy, however, that older adults who self‐report higher levels of physical activity do not have many of these purported features of ageing, highlighting inactivity as a primary mitigating factor of some of these age‐related adverse outcomes (McKendry et al., 2021).

## 5 WHAT DO WE KNOW ABOUT LOADING AND HOW IT AFFECTS PROTEOSTASIS?

The intricate interplay between internal and external variables regulates skeletal muscle proteostasis. Muscle loading, particularly resistance exercise (RE), is one of the most potent external variables leading to changes in MPS and MPB by activating/suppressing internal variables (mechano‐transduction, transcriptomic response, etc.) linked to muscle growth and loss (Lim et al., 2022). In the postabsorptive state, RE promotes an increase in both mixed MPS and MPB in untrained individuals; however, in trained individuals, while mixed MPS still increases, albeit with reduced amplitude and duration compared to untrained persons, following RE, there is no measurable change in MPB (Phillips et al., 1999). In the postprandial state, regardless of training status, MPS (mixed and subfractions measured by traditional substrate‐specific tracer infusion) increases following RE. However, MPB remains unchanged compared to resting and fasted levels, regardless of training status (Phillips et al., 2002).

Further, the reduction in MPS, assessed using D_2_O, both at rest and following RE, contributes to the loss of muscle during dietary energy restriction, with no or limited changes in MPB (Hector et al., 2018), showing that MPS actively responds to muscle loading under diverse training and nutrition conditions, as opposed to lesser responses in MPB, and primarily underpins changes in muscle mass (Figure 1). Importantly, in this study (Hector et al., 2018), the relative changes in MPS following RE were greater than that of MPB. Greater relative changes in MPS versus MPB have been shown in other studies as well. For example, although mixed MPB increased by 31% and 18% following 3 and 24 h of RE, respectively, the delta changes in MPS were 112% and 65% following 3 and 24 h of RE, respectively. Additionally, while MPB returned to the resting level after 24 h of RE, the elevated MPS following RE remained at 31% until 48 h after RE (Phillips et al., 1997). These outcomes, obtained using a stable isotope infusion, support the notion that MPS is the dominant contributor to changes in muscle proteostasis under loading conditions.

Muscle subfraction analysis shows that RE is largely associated with myofibrillar protein synthesis and less associated with mitochondrial proteins, whereas endurance exercise (EE) stimulates greater mitochondrial protein synthesis than RE (Wilkinson et al., 2008). Alongside the type of exercise, the intensity of EE or proximity to fatigue in RE sets are also important considerations, as these variables influence the amplitude of synthesis as well as the muscle subfraction‐specific response (Bagheri et al., 2022; Burd et al., 2010). As skeletal muscle is composed of an abundance of proteins, and individual proteins have variability in their turnover rate, future research should focus on analysing individual protein turnover (i.e., dynamic proteomics) using stable isotope tracers and high‐sensitivity mass spectrometry (Camera et al., 2017). Nevertheless, muscle subfraction information is robust in providing insights into the response to diverse muscle loading approaches.

## 6 WHAT DO WE KNOW ABOUT UNLOADING AND HOW IT AFFECTS PROTEOSTASIS?

Unloading through limb immobilisation, bed rest or relative disuse through abrupt reductions in physical activity results in muscle atrophy and alters muscle metabolism, particularly proteostasis (McKendry et al., 2021). For instance, muscle disuse is associated with reduced myofibrillar protein mass, primarily as a consequence of reduced myofibrillar protein synthesis at rest and in the postprandial period. Unloaded skeletal muscle also shows a robust decrease in mitochondrial transcript expression and protein abundance, as well as likely impairments in mitochondrial function (Atherton et al., 2016; McKendry et al., 2021). Interestingly, genes related to MPB are only differentially regulated after 14 days of immobilisation, with the prevailing unloaded transcriptional profile reflecting an impaired protein synthetic capacity (Abadi et al., 2009). This adaptation is exemplified by the decrease in tRNA‐synthesizing enzymes, ribosomal subunits and translation initiation factors (McKendry et al., 2021). The reduced MPS during postabsorptive and postprandial states following the period of disuse is of sufficient magnitude to account for muscle mass loss in healthy young individuals (Atherton et al., 2016) and not increases in MPB (Brook et al., 2022). Moreover, in older individuals, declines in MPS and muscle mass/volume during disuse atrophy are not always fully recoverable following a return to normal physical activity, unlike in younger adults (Shur et al., 2024).

Disuse events induce a premature ‘muscle‐full’ state (Deane et al., 2021), in which skeletal muscle becomes refractory to stimulation despite the sustained presence of elevated amino acids. This state is characterised by the blunting of fasted and fed‐state MPS measured by traditional substrate‐specific tracer infusion (i.e., anabolic resistance), leading to disturbed proteostasis akin to the age‐related phenomenon (Wall et al., 2015) and muscle atrophy that cumulatively may contribute to sarcopenia (Deane et al., 2021). Additionally, disuse induces insulin resistance and mitochondrial dysfunction (Atherton et al., 2016). The development of muscle insulin resistance during the period of disuse occurs rapidly (within 3–5 days and 3–14 days, respectively) (Atherton et al., 2016; Shur et al., 2024). This disuse‐induced muscle insulin resistance disturbs proteostasis, leading to reduced abundance and phosphorylation of key proteins involved in skeletal muscle glucose uptake, phosphorylation and storage (Bienso et al., 2012), which might mediate the disuse‐induced change in skeletal muscle turnover.

## 7 ANIMAL MODELS

**FIGURE 2:**
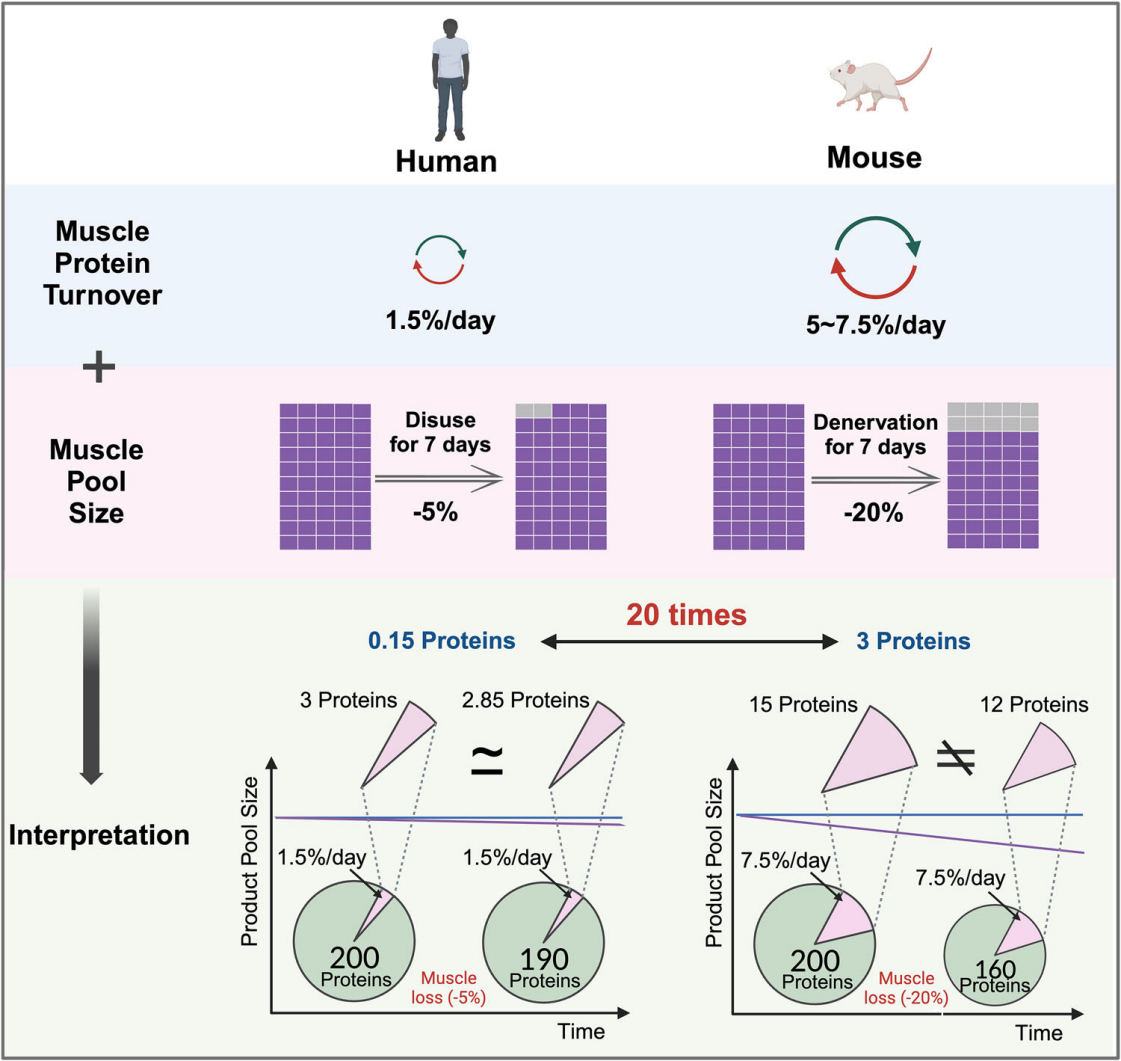
Comparison of protein turnover and muscle mass loss between human and mouse disuse models. Schematic representation of protein turnover in humans and mice showing how protein fractional turnover rates might be affected by disuse. In mice, protein turnover is approximately 5–7 times faster than in humans. Moreover, the loss of muscle mass following 7 days of denervation in mouse models is approximately 4 times greater than in common human disuse models, such as single‐leg immobilization or bed rest, over the same duration. In a simple mathematical approach, following 7 days of disuse events, there is a loss of product (i.e., muscle) pool size by 5% and 20% in humans and mice, respectively. Considering the general protein turnover rate in humans (∼1.5%/day) and mice (∼7.5%/day), a similar total protein turnover could occur between pre‐ and post‐atrophy in humans. However, the change in total protein turnover in mice between pre‐ and post‐atrophy is 20 times larger than in humans. Thus, unlike mouse models, the slower protein turnover and modest changes in pool size in humans suggest that the use of non‐steady‐state equations would not be necessary to evaluate MPS or MPB in human studies, and the physiological impact is negligible. Created with BioRender.com.

**FIGURE 3:**
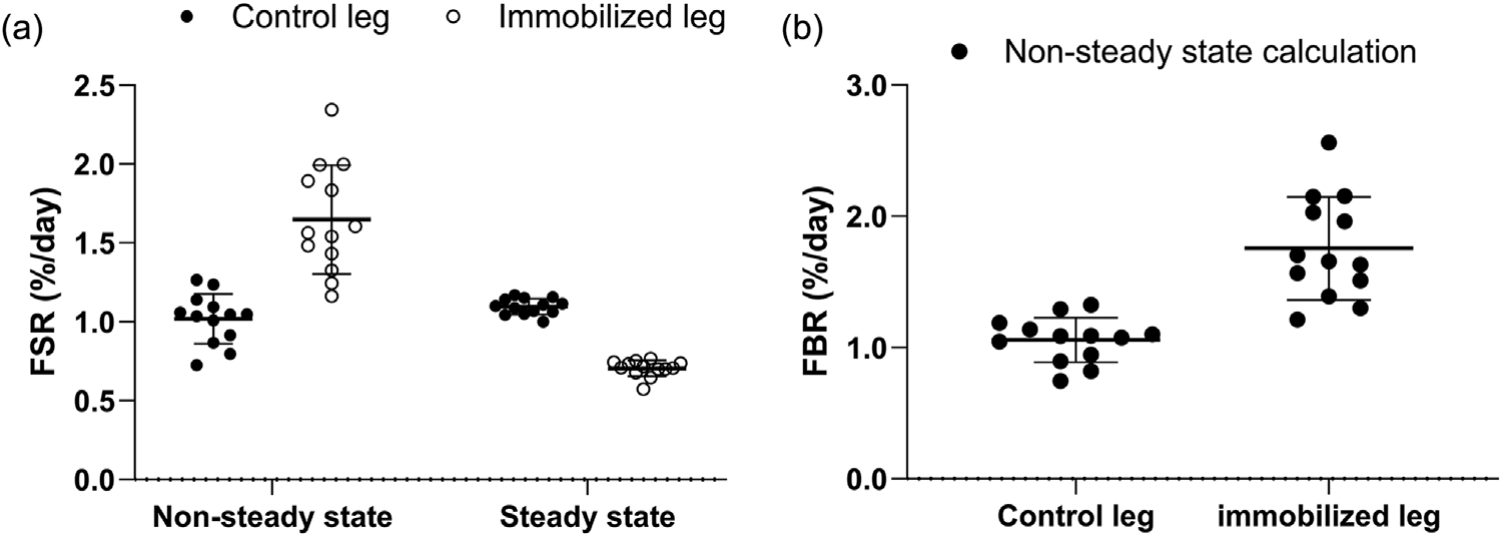
A comparison of the impact of 7 days of knee immobilization of fractional synthesis rate (FSR) in skeletal muscle, modelled using non‐steady‐state and steady‐state equations (a), and non‐steady‐state fractional breakdown rate (FBR) in the control limb and the contralateral limb after 7 days of immobilization (b). Data were taken from Kilroe et al. (2020) and modelled using non‐steady‐state equations presented in Kobak et al. (2021) and Miller et al. (2018). The following assumptions were made: muscle density is 1.04 g/cm^3^; the percentage of muscle mass that is protein is 20%; and the proportion of total muscle protein that is myofibrillar protein is 70%.

When interpreting data from animal studies, it is essential to consider the relevance of the chosen model and the physiological stimulus under investigation. While small animals like mice share similarities with humans in terms of organ systems and genetics, their physiological responses may differ significantly (Demetrius, 2005). For example, small mammals showed a reduction in skeletal muscle mass ranging from 12% in the soleus muscle to 20% in the extensor digitorum longus muscle following 7 days of hindlimb cast immobilization (You et al., 2015). Additionally, small mammals undergoing nerve dissection – a rodent model often used to induce disuse muscle atrophy – experience a 20% reduction in skeletal muscle mass in 7 days. This change represents a substantial change in the size of the protein pool, and in only 7 days, which may indeed warrant the inclusion of protein pool size differences in turnover calculations. In contrast, humans undergoing single limb immobilisation or bed rest show a 5% loss in the same period, indicating reductions in MPS and MPB (Preobrazenski et al., 2023). The loss of skeletal muscle during this period in humans can be almost entirely explained by reductions in MPS, with limited or no changes to MPB rates (Phillips & McGlory, 2014), but this is not the case in small mammals (Figure 1). Thus, extrapolating findings from small animal studies to humans should be done cautiously, considering the substantial differences in muscle mass changes and underpinning proteostasis mechanisms (Figure 2). We do not dismiss the *theoretical* advantages of employing non‐steady‐state equations to model skeletal muscle protein turnover, especially with the changes in protein pool size observed in rodents subjected to denervation. However, practical considerations limit the utility of such models in humans. Whereas the entire limb can be excised and weighed accurately in rodents (with the assumption that tissue weight changes represent changes in pool size), protein pool size estimations in humans require calculations of muscle volume, muscle density and the percentage of total muscle mass comprising protein (Figure 3 and Supplementary File 1, including the non‐steady‐state calculation R script). If muscle protein synthesis and breakdown rates are also being considered, additional assumptions and calculations are required. Each technique or biochemical assay is associated with measurement error(s), the aggregate of which is incorporated in the final measurement of protein pool size. These errors, in turn, influence non‐steady measurements of MPS (fractional synthesis rate – FSR) and MPB (fractional breakdown rate – FBR). They would contribute to the introduction of greater error into estimates of MPS and MPB (were it to be measured), as several of us have attempted to do, in comparisons to the comparatively minor changes in pool size that occur with disuse in humans relative to rodents. We have attempted (Figure 3 and Supplementary Figure 1) to use estimates and assumptions with our data to determine the results using non‐steady‐state calculations and compare the outcomes to steady‐state results. Changes in MPS, measured using traditional equations, reproducibly associate with changes in muscle size in response to various interventions that humans are subjected to; these include short‐term infusions (presumably not subject to errors in pool size) and longer‐term measures (Atherton et al., 2016; Brook et al., 2022; Phillips & McGlory, 2014); the non‐steady‐state observation that FSR is elevated seems odd in comparison (Figure 3). The elevation FBR is not conceptually incorrect but is hard to reconcile with the known rates of muscle loss in disuse and, indeed given the arrangement of the non‐steady‐state equations and the derived responses, is a direct reflection of the elevated FSR. Thus, while steady‐state and non‐steady‐state equations come with caveats, perhaps George Box had it right, stating that ‘all models are wrong, but some are useful’. In sum, it is imperative to appreciate the limitations and the positive knowledge advances in stable isotope considerations in humans and to relate such considerations to the physiological and metabolic contexts of the situations we have outlined here and also the potential for substantial differences between rodent versus human investigations.

## 8 SUMMARY

To summarise, most importantly to physiology, longer‐term integrated tracer approaches using D_2_O employing steady‐state equations align well with changes in protein mass at the tissue level in humans. Nonetheless, it remains crucial to balance the use of newer techniques alongside established methods to gain a comprehensive understanding of protein turnover. Recognising this, the use of D_2_O dynamic proteomics approaches to capture the turnover of individually identified proteins is promising. With regard to the potential role of dynamic proteomics turnover versus ostensible bulk turnover in resolving the issues identified, many of these still exist and there will be additional issues that are specific to this approach. For instance, both approaches are impacted by:

- The need to isolate proteins from specific cell types and sub‐fractions and the relative abundance of these, in addition to pH (or other techniques) for increasing coverage of the variable abundant proteins that make up these fractions.
- As outlined, whether you are measuring faster or slower protein turnover, the dosing and duration of the tracer approach require consideration; in general, fast turnover proteins require a short duration, and slower turnover proteins require a longer duration or higher dose/infusion of tracer. However, with bulk protein turnover, the contribution of these faster turning‐over proteins to the overall abundance of the bulk protein portion is a lesser consideration. Large proportions of proteins in myofibrillar or mixed proteins are made up of structural or contractile proteins, which typically have a relatively slow turnover.
- In relation to adopting a steady‐state approach, both may need to account for changes in pool size, and arguably, this has a greater potential impact with faster turnover, less abundant proteins and more so in dynamic proteomics sampling. In addition, changes in pool size would need to be substantial, as is often seen in preclinical animal models, to significantly impact tracer measures, which is less likely to occur in human models.
- Although dynamic proteomics has the potential to identify many more proteins, coverage remains limited to a few thousand proteins using even the highest resolution mass spectrometry (MS) instrumentation. It is suggested that the skeletal muscle proteome consists of ∼13,000 proteins (https://www.proteinatlas.org/humanproteome/tissue/skeletal+muscle). Even with the best equipment in the world it would not be possible to measure the turnover of all these proteins using stable isotopes, limiting the coverage of the skeletal muscle proteome. So, it is *inherently* biased for insights to exclusively use dynamic protein turnover, while bulk measures will take the mean of the whole proteome as their measure. Furthermore, measurement of the proteome, i.e., the ID of proteins or peptides, is different from having sufficient resolution to integrate the isotopomer distribution for the labelling of that protein or peptide. Therefore, despite perhaps detecting a few hundred or a few thousand proteins with MS, the number of these that can be accurately measured for turnover will be significantly less, reducing coverage even further.
- Cost and access to analytical (and idealistic work‐in‐progress mathematical and bioinformatic) expertise means that the costs are much higher for the high‐resolution mass spectrometers required to make these highly sensitive measurements such that both acquisition and running costs limit widespread usage.

In reality, both full proteome and fractional turnover approaches can co‐exist. Each process provides valuable information as to the physiology and mechanisms that underpin muscle protein turnover (proteostasis), and both still lag in making accurate and robust measures of MPB. In general, the measurement of bulk or sub‐fraction MPS has proven invaluable in understanding the impact of nutrition, exercise and hormones in health, ageing and disease and should continue to do so, with even greater understanding that may be derived from the application of dynamic proteomics. Critically, MPS remains valid irrespective of whether 10,000 or 100 proteins are being quantified or, to a considerable degree, in terms of pool size changes.

## AUTHOR CONTRIBUTIONS

All authors designed and drafted the work or revised it critically for important intellectual content. All authors have approved the final version of the manuscript and agreed to be accountable for all aspects of the work in ensuring that questions related to the accuracy or integrity of any part of the work are appropriately investigated and resolved. All persons designated as authors qualify for authorship, and all those who qualify for authorship are listed.

## CONFLICT OF INTEREST

No competing interests declared.

## FUNDING INFORMATION

No funding was received for this work.

## Supporting information



Supplementary File 1. Script for non‐steady‐state calculation in R.
Supplementary Figure 1. Fractional synthesis rate (FSR) was calculated using non‐steady‐state and steady‐state equations from tissue extracted before and after 14 days of knee immobilization (A), and non‐steady‐state fractional breakdown rate (FBR) rates in response to 14 days of knee immobilization, calculated using non‐steady‐state equations (B).
