# Supplementary material for: Turning over new ideas in human skeletal muscle proteostasis: What do we know and where to from here?
Source: Exp Physiol. 2025 Feb 5:10.1113/EP092353. Online ahead of print. doi: 10.1113/EP092353 (PMC13394054; doi:10.1113/EP092353)
Supplement: Supplementary file 1 — Script for non‐steady‐state calculation in R. Supplementary Figure 1. Fractional synthesis rate (FSR) was calculated using non‐steady‐state and steady‐state equations from tissue extracted before and after 14 days of knee immobilization (A), and non‐steady‐state fractional breakdown rate (FBR) rates in response to 14 days of knee immobilization, calculated using non‐steady‐state equations (B). [file EPH-9999-0-s001.pdf]

SUPPLEMENTAL DATA FOR

Turning Over New Ideas in Human Skeletal Muscle Proteostasis: What do We Know and Where to from Here?

Changhyun Lim.,1,2 James McKendry,1,3 Matthew Lees, 1 Philip J. Atherton,4,5 Nicholas A. Burd,6 Andrew M. Holwerda,7 Luc J.C. van Loon,7 Chris McGlory,8,9 Cameron J. Mitchell,10 Kenneth Smith,4 Daniel J. Wilkinson,4 Tanner Stokes,1 and Stuart M. Phillips1,11*

1 Exercise Metabolism Research Group, Department of Kinesiology, McMaster University, Hamilton, Ontario, Canada
2 Population Health Sciences Institute, Faculty of Medical Sciences, Newcastle University, Newcastle upon Tyne, UK
3 Food, Nutrition and Health, Faculty of Land and Food Systems, The University of British Columbia, Vancouver, BC, Canada
4 MRC/ARUK Centre for Musculoskeletal Ageing Research and National Institute of Health Research, Biomedical Research Centre, School of Medicine, University of Nottingham, Derby, UK
5 Ritsumeikan University, Ritsumeikan Advanced Research Academy (RARA) Fellow and Visiting Professor, Faculty of Sport and Health Science, Kyoto, Japan
6 Department of Health and Kinesiology and Division of Nutritional Sciences, University of Illinois Urbana-Champaign, Urbana, IL, United States
7 Department of Human Biology, NUTRIM Institute of Nutrition and Translational Research in Metabolism, Maastricht University Medical Center+, Maastricht, the Netherlands
8 School of Kinesiology and Health Studies, Queen's University, Kingston, Ontario, Canada
9 Department of Medicine, Queen's University, Kingston, Ontario, Canada
10 Faculty of Education, School of Kinesiology, The University of British Columbia, Vancouver, BC, Canada
11 Department of Sport and Exercise Sciences, Manchester Metropolitan University Institute of Sport, Manchester, UK

Supplementary File 1. Script for non-steady-state calculation in R

```
##### - Update Libraries and Setup - #####
library(dplyr)
library(ggplot2)
library(reshape2)
library(tidyr)

set.seed(12345)

##### - Create Functions - #####
generate_data <- function(n, mean, sd, min_value = 0) {
  data <- rnorm(n, mean = mean, sd = sd)
  data[data < min_value] <- min_value
  return(data)
}

# Below function makes the following assumption: total quad mass is
~20% protein, and myo is ~70% of protein = 0.2*0.7=0.14
calculate_total_protein <- function(volume_cm3, density) {
  mass_g <- volume_cm3 * density
  myofibrillar_protein_g <- mass_g * 0.14
  return(myofibrillar_protein_g)
}

##### - Initial Conditions - #####
# Unless otherwise stated, values were obtained from Kilroe et. al
2020. AJP Endo.

N <- 13
muscle_density <- 1.04  # g/cm³ # Ref: ICRP, 1975. Report of the Task
Group on Reference Man. ICRP Publication 23. Pergamon Press, Oxford

# Baseline quadriceps volumes (cm³)
control_mean_vol_baseline <- 2315
control_sd_vol_baseline <- 120
immob_mean_vol_baseline <- 2342
immob_sd_vol_baseline <- 129

# Enrichment parameters
baseline_protein_enrichment_mean <- 0.0015
baseline_protein_enrichment_sd <- 0.0005
baseline_precursor_enrichment_mean <- 2.8
baseline_precursor_enrichment_sd <- 0.1
post_precursor_enrichment_mean <- 3.5
post_precursor_enrichment_sd <- 0.1

# Post-protein enrichment
```

```
control_post_protein_enrichment_mean <- 0.2448
control_post_protein_enrichment_sd <- 0.01
immob_post_protein_enrichment_mean <- 0.1596
immob_post_protein_enrichment_sd <- 0.01

##### - Generate Data - #####
# Generate baseline and post-intervention muscle volumes
control_baseline_volume <- generate_data(N, mean =
control_mean_vol_baseline, sd = 120)
immobilized_baseline_volume <- generate_data(N, mean =
immob_mean_vol_baseline, sd = 129)

# Post-intervention muscle volumes
control_post_volume <- control_baseline_volume * generate_data(N, mean
= 1, sd = 0.01)  # Minimal change
immobilized_post_volume <- immobilized_baseline_volume *
generate_data(N, mean = 0.93, sd = 0.02)  # ~7% reduction

# Calculate total myofibrillar protein mass (g)
control_total_protein_baseline <-
calculate_total_protein(control_baseline_volume, muscle_density)
immobilized_total_protein_baseline <-
calculate_total_protein(immobilized_baseline_volume, muscle_density)
control_total_protein_post <-
calculate_total_protein(control_post_volume, muscle_density)
immobilized_total_protein_post <-
calculate_total_protein(immobilized_post_volume, muscle_density)

# Generate enrichment data
baseline_protein_enrichment <- generate_data(2 * N, mean =
baseline_protein_enrichment_mean, sd = 0.0005)
baseline_precursor_enrichment <- generate_data(2 * N, mean =
baseline_precursor_enrichment_mean, sd = 0.1)
post_precursor_enrichment <- generate_data(2 * N, mean =
post_precursor_enrichment_mean, sd = 0.1)
control_post_protein_enrichment <- generate_data(N, mean =
control_post_protein_enrichment_mean, sd = 0.01)
immobilized_post_protein_enrichment <- generate_data(N, mean =
immob_post_protein_enrichment_mean, sd = 0.01)

# Calculate average precursor enrichment
average_precursor_enrichment <- (baseline_precursor_enrichment +
post_precursor_enrichment) / 2

##### - Combine Data into Data Frame - #####
data <- data.frame(
  Subject = rep(1:N, times = 2),
  Limb = rep(c("Control", "Immobilized"), each = N),
```

```
  Baseline_Quadriceps_Volume_cm3 = c(control_baseline_volume,
immobilized_baseline_volume),
  Post_Quadriceps_Volume_cm3 = c(control_post_volume,
immobilized_post_volume),
  Total_Protein_Baseline_g = c(control_total_protein_baseline,
immobilized_total_protein_baseline),
  Total_Protein_Post_g = c(control_total_protein_post,
immobilized_total_protein_post),
  Baseline_Protein_Enrichment = baseline_protein_enrichment,
  Post_Protein_Enrichment = c(control_post_protein_enrichment,
immobilized_post_protein_enrichment),
  Baseline_Precursor_Enrichment = baseline_precursor_enrichment,
  Post_Precursor_Enrichment = post_precursor_enrichment,
  Average_Precursor_Enrichment = average_precursor_enrichment
)

##### - Calculate Non-Steady-State Metrics - #####
data <- data %>%
  mutate(
    t = 7, # Time interval (days)
    E_star = Average_Precursor_Enrichment, # Precursor enrichment
(fraction)
    E_t = Post_Protein_Enrichment, # Protein-bound enrichment
(fraction)

    # Calculate k_deg
    k_deg = -(1 / t) * log((1 - E_t / E_star) * (Total_Protein_Post_g
/ Total_Protein_Baseline_g)),
    FBR = k_deg * 100,

    # Calculate FSR
    FSR =
(100/t)*(1/Total_Protein_Baseline_g)*(Total_Protein_Baseline_g-
Total_Protein_Post_g*(1-
Post_Protein_Enrichment/Average_Precursor_Enrichment)),

    # Updated calculation of Peq using the paper's formula (Miller B
et al. 2018. J Physiol.)
    Peq = (E_t / E_star * Total_Protein_Post_g) /
      (1 - ((1 - (E_t / E_star)) * (Total_Protein_Post_g /
Total_Protein_Baseline_g))),

    # Calculate k_syn
    k_syn = k_deg * Peq,

    # Absolute degradation rate (g/day)
    Absolute_Degradation_Rate = k_deg * Total_Protein_Post_g,
```

```
    # Net protein change rate (g/day)
    Net_Change_Rate = k_syn - Absolute_Degradation_Rate
  )

write.csv(data, file="Updated_KilroeSims_Nov2024_v2.csv", row.names = 
FALSE)

##### - Summary Statistics - #####
mean_changes <- data %>%
  group_by(Limb) %>%
  summarize(
    Mean_k_deg = mean(k_deg, na.rm = TRUE),
    Mean_FSR = mean(FSR, na.rm = TRUE),
    Mean_FBR = mean(FBR, na.rm = TRUE),
    Mean_Peq = mean(Peq, na.rm = TRUE),
    Mean_k_syn = mean(k_syn, na.rm = TRUE),
    Mean_Absolute_Degradation_Rate = mean(Absolute_Degradation_Rate, 
na.rm = TRUE),
    Mean_Net_Change_Rate = mean(Net_Change_Rate, na.rm = TRUE)
  )

print(mean_changes)

##### - Visualize Results - #####
create_plot <- function(data, y_var, y_lab, title) {
  ggplot(data, aes(x = Limb, y = .data[[y_var]], fill = Limb)) +
    geom_boxplot(outlier.size = 1.5, outlier.shape = 21, alpha = 0.8, 
width = 0.6) +
    geom_jitter(aes(color = Limb), width = 0.2, size = 1.5, alpha = 
0.6) +  # Add jittered points
    stat_summary(fun = mean, geom = "point", shape = 23, size = 3, 
fill = "white") +
    labs(title = title, y = y_lab, x = "Limb") +
    scale_fill_manual(values = c("#4D9DE0", "#E15554"), labels = 
c("Control", "Immobilized")) +
    scale_color_manual(values = c("#4D9DE0", "#E15554")) +  # Match 
point colors with box colors
    theme_classic() +
    theme(
      text = element_text(size = 14, family = "sans"),
      axis.text = element_text(size = 12),
      axis.title = element_text(size = 14, face = "bold"),
      legend.position = "none",
      plot.title = element_text(size = 16, hjust = 0.5, face = 
"bold"),
      panel.grid.major = element_blank(),
      panel.grid.minor = element_blank()
    )
```

```
}

# Plotting fractional rates
create_plot(data, "FSR", "Fractional Synthesis Rate (%/day)",
"Fractional Synthesis Rate (FSR)")
create_plot(data, "FBR", "Fractional Breakdown Rate (%/day)",
"Fractional Breakdown Rate (FBR)")

# Plotting absolute rates
create_plot(data, "Absolute_Degradation_Rate", "Absolute Degradation
Rate (g/day)", "Absolute Degradation Rate")
create_plot(data, "Net_Change_Rate", "Net Protein Change Rate
(g/day)", "Net Protein Change Rate")
```

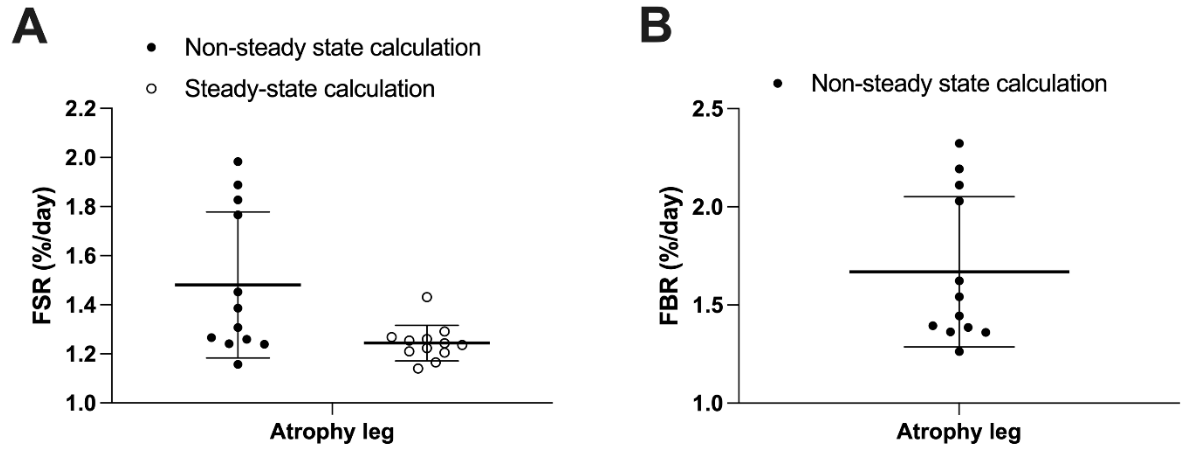

Supplementary Figure 1. Fractional synthesis rate (FSR) was calculated using non-steady state and steady-state equations from tissue extracted before and after 14 days of knee immobilization (A), and non-steady state fractional breakdown rate (FBR) rates in response to 14 days of knee immobilization, calculated using non-steady-state equations (B). Data were taken from Stokes et al. (2020) and modelled using non-steady-state equations presented in Kobak et al. (2021) and Miller et al. (2018). The following assumptions were made: muscle density = 1.04 g/cm^3; percentage of muscle mass that is protein = 20%; proportion of total muscle protein that is myofibrillar protein = 70%.

Kobak KA, Lawrence MM, Pharaoh G, Borowik AK, Peelor FF, 3rd, Shipman PD, Griffin TM, Van Remmen H & Miller BF. (2021). Determining the contributions of protein synthesis and breakdown to muscle atrophy requires non-steady-state equations. J Cachexia Sarcopenia Muscle 12, 1764-1775.

Miller BF, Hamilton KL, Majeed ZR, Abshire SM, Confides AL, Hayek AM, Hunt ER, Shipman P, Peelor FF, 3rd, Butterfield TA & Dupont-Versteegden EE. (2018). Enhanced skeletal muscle regrowth and remodelling in massaged and contralateral non-massaged hindlimb. J Physiol 596, 83-103.

Stokes T, Timmons JA, Crossland H, Tripp TR, Murphy K, McGlory C, Mitchell CJ, Oikawa SY, Morton RW, Phillips BE, Baker SK, Atherton PJ, Wahlestedt C & Phillips SM. (2020). Molecular Transducers of Human Skeletal Muscle Remodeling under Different Loading States. Cell Rep 32, 107980.
